# Impact of low intensity pulsed ultrasound on volumetric root resorption of maxillary incisors in patients treated with clear aligner therapy: A retrospective study

**DOI:** 10.1590/2177-6709.28.2.e2321252.oar

**Published:** 2023-05-29

**Authors:** Ra’ed AL-DBOUSH, Antonio ROSSI, Tarek EL-BIALY

**Affiliations:** 1 University of Alberta, Faculty of Medicine and Dentistry, School of Dentistry, Division of Orthodontics (Edmonton/Canada).; 2 Jordanian Royal Medical Services, Department of Dentistry, Division of Orthodontics (Amman/Jordan).

**Keywords:** Orthodontically-induced inflammatory root resorption, Low-intensity pulsed ultrasound, CBCT, Human

## Abstract

**Objective:**

The aim of this study was to evaluate the volumetric root resorption in maxillary incisors following clear aligner therapy (CAT) with low-intensity pulsed ultrasound (LIPUS), and compare the results to CAT alone.

**Material and Methods:**

This retrospective study evaluated pretreatment (T0) and post-treatment (T1) cone-beam computed tomography imaging of 42 adult patients. Twenty-one patients (14 females, 7 males, mean age= 38.1±12.96 years) were treated using CAT with LIPUS device, whereas the other twenty-one matching controls patients (15 females, 6 males, mean age= 35.6±11.7 years) were treated using CAT alone. Images were analyzed and a segmentation protocol was applied on the maxillary incisors. Each segmented tooth volume was exported as a surface mesh in the Visualization Toolkit (VTK) file format. The VTK files for all maxillary incisors were coded and corresponding teeth volumes from T0 and T1 were superimposed. Clipping the crown of each tooth was done, then measurements of root volumes and differences between groups were performed. Changes in root volumes were assessed (*p*<0.05).

**Results:**

Root loss was evident in all teeth in both groups, but was significantly increased in all maxillary incisors of the control group (*p*<0.001) and in upper left central incisor of LIPUS group (*p*=0.009). When both groups were compared, there was statistically significant minimal volumetric root loss in LIPUS group (3.50-7.32 mm^3^), when compared to control group (11.48-12.95 mm^3^) (*p*<0.05).

**Conclusion:**

LIPUS group showed less volumetric root resorption compared to control group during the studied treatment time using clear aligners.

## RESUMO

**Objetivo:**

O objetivo deste estudo foi avaliar volumetricamente a reabsorção radicular em incisivos superiores após tratamento com alinhadores transparentes (CAT) com e sem uso adjuvante de ultrassom de baixa intensidade (LIPUS).

**Material e Métodos:**

Esse estudo retrospectivo avaliou imagens de tomografia computadorizada de feixe cônico pré-tratamento (T0) e pós-tratamento (T1) de 42 pacientes adultos: 21 pacientes (14 mulheres, 7 homens, idade média= 38,1±12,96 anos) foram tratados com CAT e LIPUS, enquanto os outros 21 pacientes controles correspondentes (15 mulheres, 6 homens, idade média= 35,6±11,7 anos) foram tratados apenas com CAT. As imagens foram analisadas e foi aplicado um protocolo de segmentação dos incisivos superiores. Os volumes de cada dente segmentado foram exportados como malhas de superfície, em arquivos no formato *Visualization Toolkit* (VTK). Os arquivos VTK de todos os incisivos superiores foram codificados e foram sobrepostos os volumes dos dentes correspondentes a T0 e T1. Foi realizada a clipagem da coroa de cada dente e, em seguida, foram realizadas medições dos volumes radiculares e comparadas as diferenças entre os grupos, avaliando-se as alterações nos volumes de raízes (*p*<0,05).

**Resultados:**

A perda radicular foi evidente em todos os dentes em ambos os grupos, mas foi significativamente maior em todos os incisivos superiores do grupo controle (*p*<0,001) e no incisivo central superior esquerdo do grupo LIPUS (*p*=0,009). Quando ambos os grupos foram comparados, houve perda volumétrica mínima estatisticamente significativa no grupo LIPUS (3,50-7,32 mm^3^), em comparação ao grupo controle (11,48-12,95 mm^3^) (*p*<0,05).

**Conclusão:**

O grupo LIPUS apresentou menor volume de reabsorção radicular, em comparação ao grupo controle, durante o tempo de tratamento estudado usando alinhadores transparentes.

## INTRODUCTION

Root resorption is an inevitable pathologic sequela of the biological processes that occur during orthodontic tooth movement (OTM). This phenomenon is commonly known as orthodontically-induced inflammatory root resorption (OIIRR), and affects the surface of the tooth root -especially the apical part-, during which resorption of hard tissue components of the root may occurs.^1^ OTM involves several biological processes and interactions at the cellular level along the root/bone interface from cementoenamel junction (CEJ) to root apex. Inflammatory reaction to the applied force is essential for OTM, but if uncontrolled, it triggers root resorption process.^2^ The cellular mechanism of OIIRR is characterized by elevated concentrations of nuclear factor-kappa B ligand (RANKL) and reduced concentrations of osteoprotegerin (OPG).^3^ It is well known that severe OIIRR compromises the success of orthodontic treatment. The maxillary incisors are the teeth most affected by OIIRR.^4^ OIIRR is a complex multifactorial condition that is influenced by risk factors that may increase its incidence or severity: part of these factors is related to the patient, the others are related to the treatment mechanics. Patient-related risk factors includes abnormal root shape,^5^ ^-^ ^8^ racial variation,^4^ genetic predisposition,^9^ ^,^ ^10^ being asthmatic^11^ and hypodontia.^12^ Several authors reported that there was no difference in either the incidence or severity of root resorption between male and female patients.^13^ ^-^ ^15^ treatment-related risk factors includes extraction of maxillary first premolars to correct protruded teeth,^4^ ^,^ ^5^ ^,^ ^8^ moving the apices for long distance,^6^ ^,^ ^7^ lingual root torque,^16^ intrusion of incisors,^17^ ^-^ ^19^ intermaxillary elastics,^6^ force magnitude^20^ and long duration of the treatment.^16^ ^-^ ^18^ Recent adjunctive interventions that aim to reduce total treatment duration and OIIRR are the main interest for several researchers. One type of these interventions is low-intensity pulsed ultrasound (LIPUS).

Ultrasound, an acoustic pressure wave at frequencies above the limit of human hearing, is transmitted through and into biologic tissues. It has been used widely in medicine as a therapeutic, operative, and diagnostic tool.^21^ LIPUS output is of low intensity enough to be considered neither thermal nor destructive.^22^ Previous research have shown that LIPUS reduces OIIRR through two major mechanisms: the first is stimulation of cementum deposition, through stimulation of cementoblasts;^23^ and the second is inhibition of cementoclastogenesis, by altering OPG/RANKL ratio.^24^

To date, no study assessed the effects of LIPUS on volumetric root loss in teeth after clear aligner therapy (CAT) in human subjects. The objective of this retrospective study was to assess if LIPUS reduces the severity of OIIRR in maxillary incisors, as evaluated by changes in tooth root volume after CAT, compared to no LIPUS treated patients. The null hypothesis tested was that there would be no significant difference in volumetric root loss in teeth treated using CAT with LIPUS or CAT alone.

## MATERIAL AND METHODS

This was a retrospective study carried out on pretreatment (T0) and post-treatment (T1) cone beam computed tomography (CBCT) images of adult patients (age range between 18 years and 59 years) who were treated using CAT (Invisalign, Align Technology, Santa Clara, CA, USA) by the same orthodontist (TE) at his private orthodontic clinic in Edmonton, Canada, during a span of 5 years (2016-2020). CBCT images used were acquired as diagnostic records for orthodontic treatment planning. The patients had signed an informed consent form allowing the use of their data for scientific purposes. The study has been approved by the Health Research Ethics Board at the University of Alberta, Canada (Pro00091339). Data about sex, age, treatment duration and total number of aligners for each patient were also collected (Table 1). During the orthodontic treatment planning stage, patients included in this study were instructed with information about tooth movement accelerating methods, in the form of brochures, videos and personalized discussions. The decision to use the LIPUS device or not was done by the patient and his/her family depending on their desire to shorten the treatment time and affordability for the extra cost of the adjunctive device. LIPUS was applied to the intervention group using an ultrasound device (Aevo system, SmileSonica Inc., Edmonton, AB, Canada) concurrently with CAT. The LIPUS device was used by the patient at home for 20 min/day during the whole treatment, with the parameters shown in Appendix 1. The other group, which served as a control group, was treated using CAT alone. The usage protocol of aligners in the intervention group was to change the aligners every 5 days, while the usage protocol of aligners in the control group was to change the aligners every 7 to 10 days.

**Table 1:** Characteristics of included patients in both groups, with inclusion and exclusion criteria.

	LIPUS (n=21)	Control (n=21)	*p-* value*
Age (years) - Mean ± SD	38.1 ± 12.96	35.6 ± 11.7	0.535^‡^
Males (n)	7	6	0.739^†^
Females (n)	14	15	0.739^†^
Aligners (trays) - Mean ± SD	79.3 ± 24.1	91.2 ± 26	0.134^‡^
Little’s irregularity index - Mean ± SD	3.8 ± 3.35	3.75 ± 1.4	0.918^‡^
ABO discrepancy index - Mean ± SD	17.9 ± 6.54	17.76 ± 5.82	0.94^‡^
Treatment duration (months) - Mean ± SD	15.82 ± 5.05	27.79 ± 9.5	<0.001^*‡^
Class II malocclusion (n)	12	12	NA
Class III malocclusion (n)	9	9	
» Inclusion criteria: - Adult patients who were treated using clear aligner therapy - Availability of pretreatment and post-treatment cone beam computed tomography images - Mild to moderated crowding (0-6 mm), based on the Little’s irregularity index of maxilla - Class II or III molar relationship - No history of orthodontic treatment - No periodontal diseases - No significant medical history - Not asthmatic - No craniofacial anomalies - No missing teeth - No history of trauma to the maxillary incisors - Non-extraction treatment and non-surgical treatment			
» Exclusion criteria: - History of previous trauma or endodontic treatment - Patients with severe crowding (>7mm) in the anterior area - Pregnant women - Systemic conditions that may affect root resorption, like asthma - Chronic use of medications affecting orthodontic tooth movement, as bisphosphonate - History of parafunctional habits			

^*^ Statistically significant (*p*<0.05). ^‡^ Independent samples student’s t-test. ^†^ Pearson’s chi-squared test.

**Figure 1:**
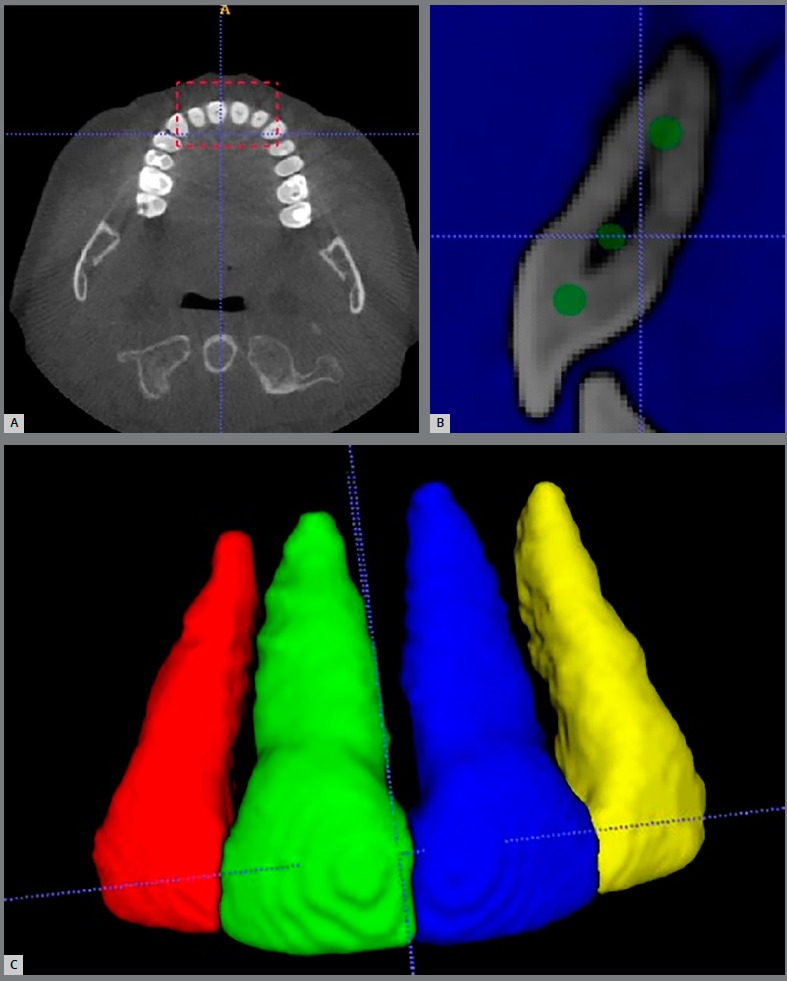
ITK-SNAP software. **A,** B) Steps of the semi-automatic 3D segmentation wizard. C) Color label for each tooth.

**Figure 2:**
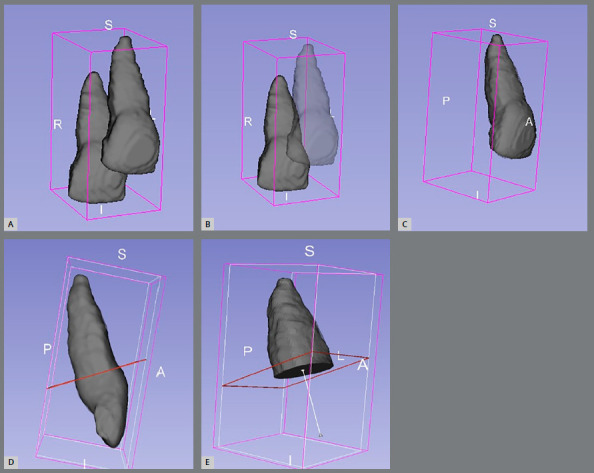
Slicer software. **A-**C) Superimposition of T0 and T1 tooth volumes. **D,**E) Clipping the crown of the merged model at the cementoenamel junction, to obtain the root volumes.

Sample size calculation was performed with G*Power v. 3.1.9.2 software, based on an alpha level of significance of 0.05 and a beta of 0.2, to achieve a power (1-b) of 0.8, assuming a large effect size difference (0.8) between groups. The results showed that a minimum of 21 patients was necessary in each group. Records were collected retrospectively, based on the detailed inclusion and exclusion criteria shown in Table 1. A control group who had been treated using CAT only was randomly selected to match the LIPUS group for age, gender distribution, baseline malocclusion, number of aligners, Little’s irregularity index (mild to moderate crowding) and ABO discrepancy index (DI) (score 7 to 32) (as measured using OrthoCad^®^ software, Cadent, Inc, Fairview, NJ, USA). The latter is considered a measure of case complexity index that evaluates the common elements of an orthodontic diagnosis: overjet, overbite, anterior open bite, lateral open bite, crowding, occlusion, lingual posterior crossbite, buccal posterior crossbite, ANB angle, IMPA, and SN-GoGn angle.^25^ The LIPUS group comprised 21 subjects (mean age 38.1±12.96 years, 7 males and 14 females). The control group comprised 21 patients (mean age 35.6±11.7 years, 6 males and 15 females). CBCT images were taken at T0 and T1 using the same imaging device (i-CAT™, Imaging Sciences International; Hatfield, Pennsylvania, USA), using the CBCT specifications shown in Appendix 1. All subjects were provided with a protective lead apron. Images were converted to Digital Imaging and Communications in Medicine (DICOM) format using the InVivo software (Anatomage, San Jose, CA). The DICOM data were then imported into the ITK-SNAP v. 3.8 software (www.itksnap.org), which is an open-source 3D medical imaging software that allows for the segmentation of structures from CBCT images.^26^ After loading the DICOM data in the ITK-SNAP, a special tool called “Active Contour Segmentation Mode’’ was used to select the area of interest (i.e., anterior maxilla) (Fig 1A). Following the selection of anterior part of the maxilla, 3-steps semi-automatic 3D segmentation wizard was started. The first step of the wizard is called pre-segmentation thresholding, being composed of two thresholds that helps the observer to visualize the image better by changing the values of gray scale: the maximum value of the upper thresholds was used, in order to include all the radiopaque structures; and the lower threshold was manipulated to obtain the most suitable gray value that showed good anatomy of the incisors. The next step was to place the baseline bubbles inside the maxillary incisors, which served as initiators for building up the 3D shape of the teeth (Fig 1B). In the final step, which is called “evolution”, actual contour segmentation was initiated, proceeded automatically in a stepwise fashion and manually stopped when the whole area of interest was covered by the colored labeling. Then, manual refinement of the teeth segmentation was undertaken using the Paintbrush mode on 2D image basis, to remove any voxels that represented surrounding anatomical structures (like bone plates or teeth other than maxillary incisors), and add any voxels that had been unintentionally omitted from the tooth volume during the semi-automatic segmentation process. Regarding the pulp cavity and canals, they were included during the manual refinement stage, to obtain the intact tooth and root volume. All refinements were performed on the multi-planar reformatted images, in axial, coronal, and sagittal orientation. After obttaining a 3D image of the four maxillary incisors as one labeled color, the scalpel tool was used in the 3D view screen, to assign a special color for each tooth. Once a special color was assigned for each tooth (Fig 1C), the “volumes and statistics” option was used to record the volume for each colored tooth structure in cubic millimeters (mm^3^). Then, each tooth was exported as a surface mesh in the Visualization Toolkit (VTK) file format. VTK files corresponding to T0 and T1 for each tooth were coded then imported together into 3D slicer v. 4.10.2 software (www.slicer.org),^27^ in which they were superimposed by the best-fit alignment, using an iterative closest point algorithm/surface registration tool (Figs 2A-2C). Using the Easy Clip module, a reference plane was constructed on the merged model between the highest point of the labial and palatal CEJ, to clip the crown and preserve the roots only (Figs 2D, 2E). The volumes of the roots were analyzed using the Models option in the main menu, and the change between the two volumes can be interpreted as increasing or decreasing the volume, i.e. as cementum loss or addition. The percentage of root volume resorption was calculated as follows: (Difference between the two volumes for a specific tooth / root volume at T0) * 100%. The severity of volumetric root resorption was classified based on the percentage of root resorption, as: mild (10%), moderate (10%-20%), and severe (20%). Statistical analysis was performed using Statistical Package for Social Sciences, v. 25.0 (SPSS for Windows, SPSS Inc., Chicago, Illinois) at the significance level of *p* <0.05. The Shapiro-Wilk test was used to verify normal distribution of the data. Paired-sample Student’s *t*-test was performed to detect significant volumetric root loss within each group. Independent samples Student’s *t*-tests were used to test for differences among all cases in both treatment groups. To test intraobserver reliability and method error, 12 randomly selected teeth were selected for re-measurement four weeks after the first measurements, by the same researcher (RA), who was blinded to treatment group assignment and did not participate in treatment of the patients. A high intraobserver reliability was found (Intraclass Correlation Coefficient = 0.942 to 0.999), as shown in Appendix 2. The method error calculated using Dahlberg’s formula ranged between 2.78 and 3.71mm^3^.^28^

## RESULTS

Characteristics of included patients in both groups are shown in Table 1. The mean ages of patients in the LIPUS and control groups at the start of the treatment were 38.1±12.96 and 35.6±11.7 years, respectively. Baseline types of malocclusions were equally distributed between both groups. No statistically significant difference was found between the groups for age, average number of aligners used, Little’s irregularity index and ABO discrepancy index (DI), using independent *t*-tests; or for sex distribution, using Pearson’s chi-squared test - indicating the homogeneity of study subjects between the LIPUS and the matched control groups at the start of treatment. All patients achieved Angle’s molar Class I and canine Class I relationships at the end of treatment.

### TREATMENT CHANGES FROM T0 TO T1 WITHIN CONTROL GROUP

The mean volumes for the roots in the control group at T0 and T1 are shown in Table 2. According to the results of paired-sample Student’s *t*-test, there was statistically significant mean root volume loss in the four incisors of the control group. The mean root volume loss ranged from 11.48 to 12.95 mm^3^, which represent 5.41 to 7.01% volumetric structure loss of the original root volume at T0. The maxillary right central incisor showed the highest mean root volume loss, while the maxillary right lateral showed the least mean root volume loss. 

**Table 2:** Mean root volumes of the maxillary incisors in the control group at T0 and T1.

Tooth	Group	Mean root volume at T0 ±SD (mm^3^)	Mean root volume at T1 ±SD (mm^3^)	Mean difference T1-T0 (mm^3^)	SD of the mean difference	P value
Right lateral incisor	Control	179.08 ± 29.76	167.59 ± 29.13	-11.48	5.09	< 0.001*
Right central incisor	Control	236.24 ± 48.34	223.28 ± 47.12	-12.95	9.07	< 0.001*
Left central incisor	Control	231.08 ± 46.9	218.30 ± 44.9	-12.78	11.68	< 0.001*
Left lateral incisor	Control	169.55 ± 25	157.68 ± 24.6	-11.87	7.48	< 0.001*

T0: Pretreatment. T1: Post-treatment. SD: Standard deviation. *Statistically significant (*p*<0.05) based on paired *t*-tests.

### TREATMENT CHANGES FROM T0 TO T1 WITHIN LIPUS GROUP

The mean volumes for the roots in the LIPUS group at T0 and T1 are shown in Table 3. According to the results of the paired-samples Student’s *t*-test, there was no statistically significant mean root volume loss in maxillary right lateral incisor (*p*=0.124), maxillary right central incisor (*p*=0.095) and maxillary left lateral incisor (*p*=0.192), while maxillary left central incisor showed significant mean root volume loss (*p*=0.009). The mean root volume loss ranged from 3.50 to 7.32 mm^3^, which represent 2.17 to 3.23% volumetric structure loss of the original root volume at T0. The maxillary left central incisor showed the highest mean root volume loss, while the maxillary left lateral incisor showed the least mean root volume loss.

**Table 3:** Mean root volumes of the maxillary incisors in the LIPUS group at T0 and T1.

Tooth	Group	Mean root volume at T0 ±SD (mm^3^)	Mean root volume at T1 ±SD (mm^3^)	Mean difference T1-T0 (mm^3^)	SD of the mean difference	P value
Right lateral incisor	LIPUS	162.32 ± 30.85	158.65 ± 31.41	-3.67	10.47	0.124
Right central incisor	LIPUS	222.97 ± 51.52	217.94 ± 52.01	-5.03	13.16	0.095
Left central incisor	LIPUS	226.61 ± 55.55	219.29 ± 55.62	-7.32	11.66	0.009*
Left lateral incisor	LIPUS	161.03 ± 27.55	157.53 ± 24.24	-3.50	11.86	0.192

T0: Pretreatment. T1: Post-treatment. LIPUS: Low-intensity pulsed ultrasound. SD: Standard deviation. *Statistically significant (*p*<0.05) based on paired *t*-tests.

### PRE AND POST-TREATMENT COMPARISON BETWEEN BOTH GROUPS

**Figure 3:**
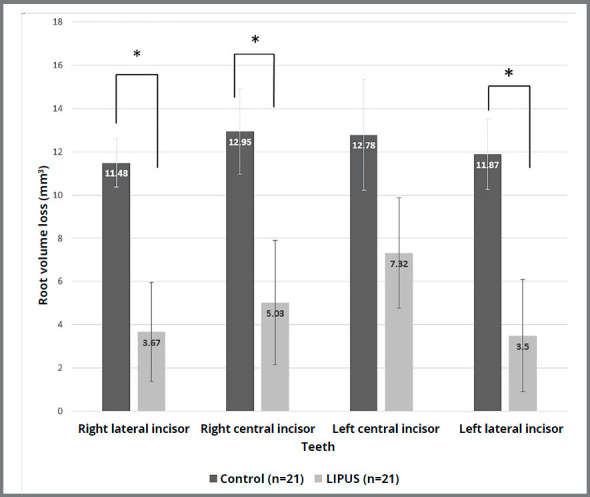
Comparison of control and LIPUS groups, regarding the mean root volume loss in maxillary incisors. Error bars represent standard error of the mean. * Statistically significant (P<0.05).

**Figure 4:**
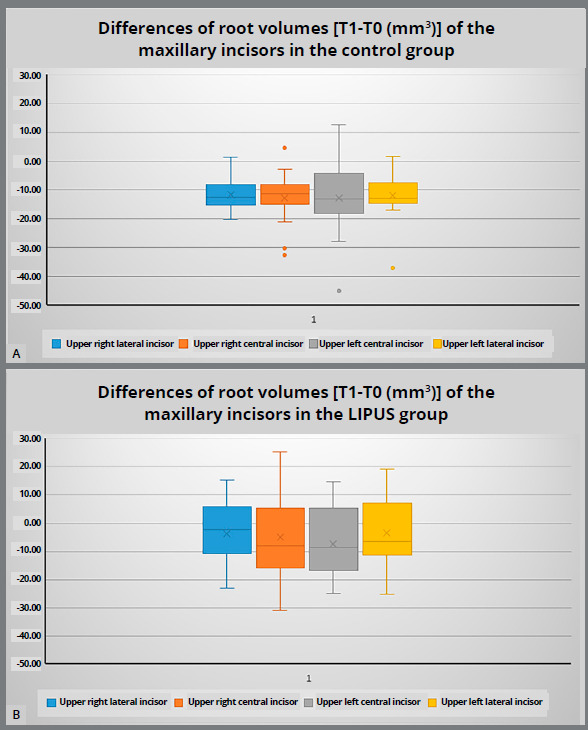
Box plot diagram showing the differences in root volumes [T1-T0 (mm^3^)] of the maxillary incisors in the control **(**A) and LIPUS**(**B) groups.

Overall, the mean root volumes for all incisors in both groups were decreased at the end of orthodontic treatment (Table 4). Results of comparison between the groups according to analysis of variance (independent samples Student’s *t*-tests) (Fig 3) showed that teeth of LIPUS group had statistically significant lower mean root volume loss, compared to the control group. However, the difference was statistically significant in maxillary right lateral incisor (*p*=0.004), maxillary right central incisor (*p*=0.029) and maxillary left lateral incisor (*p*=0.009), while there was no statistically significant difference between both groups in maxillary left central incisor (*p*=0.137). The LIPUS group had statistically significant lower percentage loss of the original root volume structure, compared to the control group (Appendix 3). Differences in root volumes [T1-T0 (mm^3^)] of the maxillary incisors in the control and LIPUS groups are shown as Box plot diagrams in Figure 4. Classification of the severity of the root volume loss in both groups is shown in Table 5. Intervention group showed less moderate resorption than control group, with no reported severe root resorption. Regarding the treatment duration, the results showed that cases treated using CAT with LIPUS had finished treatment with shorter overall time, compared to those treated using CAT alone (15.82±5.05 vs 27.79±9.5 months, respectively). The differences in treatment duration between the two groups were statistically significant (Table 1).

**Table 4:** The mean root volume loss in the maxillary incisors at T0 and T1 in both groups.

Teeth	LIPUS group (n=21) T0-T1 root volume loss Mean ±SD (mm^3^)	Control group (n=21) T0-T1 root volume loss Mean ±SD (mm^3^)	*p* **-value**
Right lateral incisor	-3.67 ± 10.47	-11.48 ± 5.09	0.004*
Right central incisor	-5.03 ± 13.16	-12.95 ± 9.07	0.029*
Left central incisor	-7.32 ± 11.66	-12.78 ± 11.68	0.137
Left lateral incisor	-3.50 ± 11.86	-11.87 ± 7.48	0.009*

T0: Pretreatment. T1: Post-treatment. LIPUS: Low-intensity pulsed ultrasound. SD: Standard deviation. *Statistically significant (*p*<0.05) based on independent samples Student’s *t*-tests.

**Table 5:** Classification of the severity of the root volume loss (volumetric root resorption) in the maxillary incisors for both groups.

Classification	Control (%)	LIPUS (%)
Mild (10 %)	84.5	91.6
Moderate (10-20 %)	14.3	8.3
Severe (more than 20%)	1.2	0

## DISCUSSION

The aim of this retrospective study was to assess the possible effect of LIPUS on OIIRR during CAT using analysis of CBCT images at T0 and T1, and to assess the treatment efficiency of using LIPUS as an adjunctive to CAT. 

The results of this study showed that LIPUS reduced mean root volume loss in maxillary incisors: the average mean root volume loss in LIPUS group ranged from 3.50 to 7.32 mm^3^, while in control group ranged from 11.48 to 12.95 mm^3^. Although the differences between the groups may be clinically irrelevant, they were statistically significant on all maxillary incisors except upper left central incisor, which could be attributed to individual variations. The LIPUS group showed less moderate root resorption (8.3%) when compared to the control group (14.3%). Also, none of the teeth in LIPUS group showed severe root resorption, while one tooth (1.4%) of the control group showed severe root resorption. The findings of this study were consistent with results of previous studies showing that application of LIPUS reduced OIIRR in different types of tooth movement.^29^ ^-^ ^32^ LIPUS reduces OIIRR mainly through stimulating cementoblasts and inhibiting cementoclasts simultaneously.^23^ ^,^ ^24^ Al-Daghreer et al^30^ investigated the effect of LIPUS on OIIRR in premolars of beagle dogs, and found that LIPUS reduced OIIRR and induced the formation of a thicker cementum and reparative cellular cementum. El-Bialy et al^29^ showed that 4-weeks LIPUS application on human premolars while applying tipping orthodontic movement decreased the areas of resorption and the number of resorption lacunae on these teeth, also healing of the resorbed root surface by hypercementosis was reported. Torque is considered one of the risk factors for OIIRR. Raza et al^31^ found that LIPUS minimized root resorption when applied during torque tooth movement over a 4-week period. A recently published randomized controlled clinical trial showed that LIPUS application accelerated tooth movement and minimized OIIRR at the same time during canine retraction.^32^ The results of the LIPUS group (Table 3) showed large standard deviations, which may be due to patients’ variability. Indeed, some teeth in the LIPUS group showed increase in the root volume at the end of treatment, as shown in Figure 4B. This was consistent with the results of previous studies^23^ ^,^ ^24^ ^,^ ^30^ showing that LIPUS increases cementum formation, and this could have contributed to healing of root resorption with more cementum than the original shape of the teeth.

Regarding the total number of aligners used, there was no statistically difference between the groups. The mean number of aligners used in the LIPUS group was 79.3±24.1, while the mean number of aligners used in the control group was 91.2±26. Hypothetically, if all patients in both groups used the same aligner wear time protocol (i.e., wearing each aligner for 1 week to 10 days, based on the aligner’s manufacturer instructions), the 12 aligner’s difference between the groups would equal a difference of three months. Indeed, the LIPUS group applied a shorter wear time per aligner (accordingly, the patients were instructed to change the aligners every 5 days), which resulted in statistically significant 12 months shortening of orthodontic treatment duration in LIPUS group (15.82±5.05 months), compared to control group (27.79±9.5 months). To understand how the LIPUS group showed this reduction in total orthodontic treatment, we should compare the control group to a hypothetical LIPUS group (Appendix 4) that would use the same number of aligners of the actual LIPUS group but with the aligner wear time of the control group. In this case, if the patients in this hypothetical group used an average of 79 aligners and changed the aligners every 7-10 days, their total treatment would be reduced by 84 to 120 days or 13%, compared to the control group, which could be not significant. However, in the actual LIPUS group, the patients used an average of 79 aligners and changed the aligners every 5 days, which led to reduction in the total treatment duration by 358 days, or 43%, compared to the control group. As shown above, the aligner wear time protocol led to significant reduction in the total duration. The reason behind applying this protocol to the LIPUS group was that the patients in this group achieved the predicted tooth movement faster than those in the control group (which could be interpreted as the LIPUS ability to move the teeth faster). The results of this study were consistent with findings of previous studies by El-Bialy et al^32^ and Kaur et al.^33^ The latter found that patients who used LIPUS showed a clinically significant reduction in the overall orthodontic treatment duration, compared to the control group, which used clear aligners only.^33^ LIPUS enhances alveolar bone remodeling, which could explain the shortened orthodontic treatment duration.^34^

From the above-mentioned observations, this study found that LIPUS reduced mean root volume loss (i.e. root resorption) in maxillary incisors, based on the analysis of the CBCT images. However, this finding should not be justified solely by the direct LIPUS preventive or reparative effect on the root cementum, as there is indirect root resorption inhibition through the reduction of treatment duration in the LIPUS group (i.e., the applied orthodontic force). The fact that the aligners were changed more frequently in LIPUS group (aligner wear time of 5 days) than in the control group (aligner wear time ranging from 7 to 10 days) resulted in reducing the treatment duration by 43%, which accordingly could help in reducing the OIIRR in the LIPUS group. In summary, the treatment duration is a confounding factor that could affect the main outcome (root resorption) of this study. Hence, the results of this study should be interpreted carefully, taking this into consideration. It is advisable that future studies should control this confounding factor by applying the same aligners wear protocol to the intervention and control groups. 

A limitation of this study was that the assessment of the outcomes was undertaken on a sample that did not include cases needing extractions. These cases are more susceptible to root resorption after moving the teeth to close the extraction spaces. The large field of view (FOV = 16x16 cm) and the voxel size of 0.3 mm were also considered limitations for this study. However, they are the most commonly used parameters for cone beam computed tomography imaging for routine orthodontic treatment planning. Due to the retrospective nature of the study, this was the best available imaging data. It is well known that as the FOV increases, the scatter levels increase,^35^ which leads to greater imaging noise,^36^ and causes reduction in the spatial resolution^35^ (i.e., the ability of the CBCT image to discriminate objects of different densities in close proximity).^37^ Although it has been reported that the two most common voxel sizes used in orthodontics are 0.3 mm and 0.4 mm,^35^ the results of one study showed that, for high precision volume measurements to be assessed accurately in-vivo, it would be better to choose a voxel size of 0.25 mm or less, as this makes the root segmentation process easier and increases the accuracy of the volume measurements obtained. However, better image quality requires a higher radiation dose and a longer scanning time.^38^ Due to the retrospective nature of this study, other limitations would include the potential biases that could result from the patient’s own decision on the group allocation due to additional treatment costs of using the LIPUS device, and the selection process of matched controls. Future randomized clinical trials can address these limitations. Finally, the lack of regression analysis test evaluating all variables that could affect root resorption and the lack of patient compliance reports of wearing the LIPUS device, that were not recorded, may be considered additional limitations for this study.

Future studies with larger study sample, images with smaller FOV, and randomized design that includes extraction cases could validate the method that was used in this study by evaluating the volumetric OIIRR before extraction using the method mentioned above and compare it the volumetric OIIRR after extraction, by scanning the extracted teeth with a desktop micro-CT machine. 

## CONCLUSIONS

The null hypothesis of this study was rejected. Within the limits of this study, LIPUS daily use for 20 minutes could result in reduction of OIIRR extent when used in conjunction with CAT. In general, this result may be related to the LIPUS capability of prevent or repair OIIRR and to the LIPUS-induced reduction of treatment time (which is a known risk factor for OIIRR).

## Appendix 1

[Table t6]

**Table t6:** Specifications of the CBCT imaging device and the LIPUS device.

Device	Specifications
CBCT imaging device	16 cm width × 16 cm height, 120 kVp, 5 mA, 3.7s scan time and 0.3-mm voxel size.
LIPUS device	Ultrasonic pulse frequency of 1.5 MHz, a pulse duration of 200 µs, a pulse repetition rate of 1 kHz (=1 ms) and spatial average-temporal average (SATA) intensity of 30 mW/cm^2^.

CBCT: Cone beam computed tomography.

LIPUS: Low-intensity pulsed ultrasound.

## Appendix 2

[Table t7]

**Table t7:** Intra-observer intraclass correlation coefficient and Dahlberg’s method error.

Variable	unit	Mean of first measurement (m1)	Mean of second measurement (m2)	Mean of absolute difference m1-m2	Dahlberg’s error	Intraclass Correlation	95% CI Upper Bound	95% CI Lower Bound
Tooth volume at T0	mm^3^	485.76±112.5	483.1±113.9	5.01	3.71	0.999	0.997	1.000
Tooth volume at T1	mm^3^	468.97±117.8	465.77±118.4	4.77	3.58	0.999	0.998	1.000
Root volume at T0	mm^3^	203.18±39.22	201.69±40.18	3.85	3.38	0.993	0.975	0.998
Root volume at T1	mm^3^	190.19±37.33	188.77±40.72	3.51	2.92	0.995	0.983	0.999
Root volume change (Root volume T1-Root volume T0)	mm^3^	-12.99	-12.93	3.80	2.78	0.942	0.812	0.983

T0: Pretreatment. T1: Post-treatment. CI: Confidence interval. mm^3^: cubic millimeter.

## Appendix 3

[Table t8]

**Table t8:** The mean percentage of root volume loss in the maxillary incisors at T0 and T1 in both groups.

Teeth	T0-T1 root volume loss (%) LIPUS group (n=21) Mean ±SD	T0-T1 root volume loss (%)Control group (n=21) Mean ±SD	P value
Right lateral incisor	-2.26±6.6	-6.51±3.1	0.009*
Right central incisor	-2.26±6.4	-5.5±3.7	0.043*
Left central incisor	-3.23±5.9	-5.41±5.1	0.181
Left lateral incisor	-2.17±7.6	-7.01±4.6	0.008*

T0: Pretreatment. T1: Post-treatment. LIPUS: Low-intensity pulsed ultrasound. SD: Standard deviation. *Statistically significant (*p*<0.05) based on independent samples Student’s *t*-tests.

## Appendix 4

[Table t9]

**Table t9:** Interpretation of the relationship between the aligner wear time protocol and the total treatment duration.

	Number of aligners Mean (SD)	Aligner wear time (days)	Expected treatment duration (days) based on number of aligners and aligner wear time*	Expected shortening of orthodontic treatment (days)	Actual treatment duration (days) based on the study results	Actual shortening of orthodontic treatment (days)	Shortening of orthodontic treatment %
LIPUS group	79 (24)	5	395	242 to 515	475	358	43%** actual
Hypothetical LIPUS group	79 (24)	From 7 to 10 days	553-790	-84 to 120	-	-	13%** hypothetical
Control group	91 (26)	From 7 to 10 days	637-910	-	833	-	-

* Expected treatment duration = Average number of aligners *x* Aligner wear time.

**Actual shortening of orthodontic treatment = (Actual treatment duration in control group - Actual treatment duration in LIPUS group) / Actual treatment duration in control group = (833-475) / 833 = 43%.

**Hypothetical shortening of orthodontic treatment in case of 7 days aligner wear = (Expected treatment duration in control group - Expected treatment duration in hypothetical LIPUS group) / Expected treatment duration in control group = (637-553) / 637 = 13%.

** Hypothetical shortening of orthodontic treatment at 7 days changing = (Expected treatment duration in control group - Expected treatment duration in hypothetical LIPUS group) / Expected treatment duration in control group = (910-790) / 910 = 13%.
